# Preclinical studies of a PARP-targeted theranostic radiopharmaceutical for pancreatic cancer

**DOI:** 10.3389/fphar.2025.1576587

**Published:** 2025-05-15

**Authors:** Jie Gao, Yuhao Wang, Ruoqi Wang, Mengya Liu, Hongliang Wang, Jianguo Li, Jin Du

**Affiliations:** ^1^ China Institute of Atomic Energy, Beijing, China; ^2^ China Institute for Radiation Protection/National Atomic Energy Agency Nuclear Technology (Nonclinical Evaluation of Radiopharmaceuticals) Research and Development Center/CNNC Key Laboratory on Radiotoxicology and Radiopharmaceutical Preclinical Evaluation, Taiyuan, Shanxi, China; ^3^ First Hospital of Shanxi Medical University, Taiyuan, Shanxi, China; ^4^ China Isotope and Radiation Corporation, Beijing, China

**Keywords:** poly ADP-ribose polymerase inhibitor, radioactive probes, pancreatic cancer, preclinical studies, radiopharmaceutical

## Abstract

**Objective:**

This study aims to improve the biodistribution of probes and enhance tumor targeting through ^68^Ga/^177^Lu-labeled optimized probes, thereby providing better tumor detection and assessment in PET imaging while also exploring their therapeutic effects on tumors.

**Methods:**

The physicochemical properties of PARPi probes were optimized through polyethylene glycol (PEG) modification. The tumor inhibition effect of the novel probes was validated through the assessment of *in vitro* affinity, uptake, *in vivo* distribution, and tumor targeting of the PARPi probes. Based on the distribution results, OLINDA/EXM radiation dose estimation was then performed to optimize the clinically administered dose.

**Results:**

In the study, a novel PARP-targeted imaging agent, DOTA-PEG-PARPi, was designed and optimized, demonstrating sufficient *in vivo* stability. The results of *in vitro* trials showed strong affinity and uptake of PEG-PARPi in pancreatic cancer tumor cells. SPECT/CT imaging revealed significant radioactive accumulation, notable uptake, and prolonged retention time in PSN-1 tumors. Tissue distribution results showed that tumor uptake peaked 3 h after administration. According to dose estimation, the highest absorbed dose was observed in the pancreas of female adults.

**Conclusion:**

The PEG-modified PARPi probe not only retained high affinity and targeting capability but also significantly improved retention time during *in vivo* trials.

## Introduction

Genomic instability in tumors is significantly increased due to endogenous and exogenous factors, such as breast cancer 1/breast cancer 2 (*BRCA1/BRCA2*) gene mutations and oxidative stress (Chen et al., 2022). Among various DNA repair factors, the overexpression of poly ADP-ribose polymerase-1 (PARP-1) family members in tumor cells has drawn particular attention (Ke et al., 2022). PARP-1 plays a crucial role in identifying DNA damage and maintaining genomic integrity (Safdar et al., 2024; Steffen et al., 2014). The inhibition of PARP-1 expression can disrupt DNA repair and replication processes in cancer cells, making them more susceptible to death (Bhamidipati et al., 2023; Soung and Chung, 2023). The United States Food and Drug Administration (FDA) has approved multiple PARP inhibitors for the treatment of BRCA mutation-positive breast cancer (Mccann and Hurvitz, 2018) and advanced ovarian cancer (Colombo et al., 2018). Nevertheless, current studies on the application of PARP-1-targeting probes in tumor therapy remain limited, necessitating further research, exploration, and validation of their potential clinical value (Li et al., 2023).

Due to the extensive application of PARP inhibitors in the treatment of ovarian and breast cancer, PARP imaging technology holds promise for providing more optimized treatment options for patients with these cancers (Slade, 2020; Cortesi et al., 2021). Recently, several research teams have demonstrated the potential of ^18^F-labeled PARP inhibitors in PARP imaging through preclinical studies (Reiner et al., 2011; Zhou et al., 2018; Mcdonald et al., 2021). Most of these compounds share structural similarities with olaparib and have shown positive efficacy in the treatment of breast and pancreatic cancers. However, the current ^18^F-labeled PARP inhibitors are primarily metabolized by the liver and excreted via bile, which potentially cause radioactive accumulation in the liver and other abdominal organs, thereby affecting image quality and hindering lesion identification, particularly at metastatic sites commonly observed in pancreatic and ovarian cancers (Young et al., 2024). The inhibitors encounter resistance challenges similar to those faced by other targeted anticancer drugs. Some patients even exhibit primary resistance at the early stages of treatment, significantly limiting the clinical application of PARPis (Young et al., 2024; Wang et al., 2023). To overcome these limitations, this study explored new labeling strategies, improved chemical properties of compounds, and developed more effective PARP inhibitors to provide better tumor detection and assessment in PET imaging. The utilization of PARP-1-targeting probes to detect PARP-1 expression in tumor cells has potential value for identifying patient populations suitable for PARPi-targeted therapy and improving treatment efficacy (Dibitetto et al., 2024; Bhin et al., 2023; Wang et al., 2019). Furthermore, such probes play a vital role in predicting therapeutic responses and quantifying tumor treatment outcomes. Therefore, it is essential to develop methods for evaluating PARP expression and activity to help identify patients who may benefit from these treatments.

## Materials and methods

### Synthesis and radiolabeling

**FIGURE 1:**
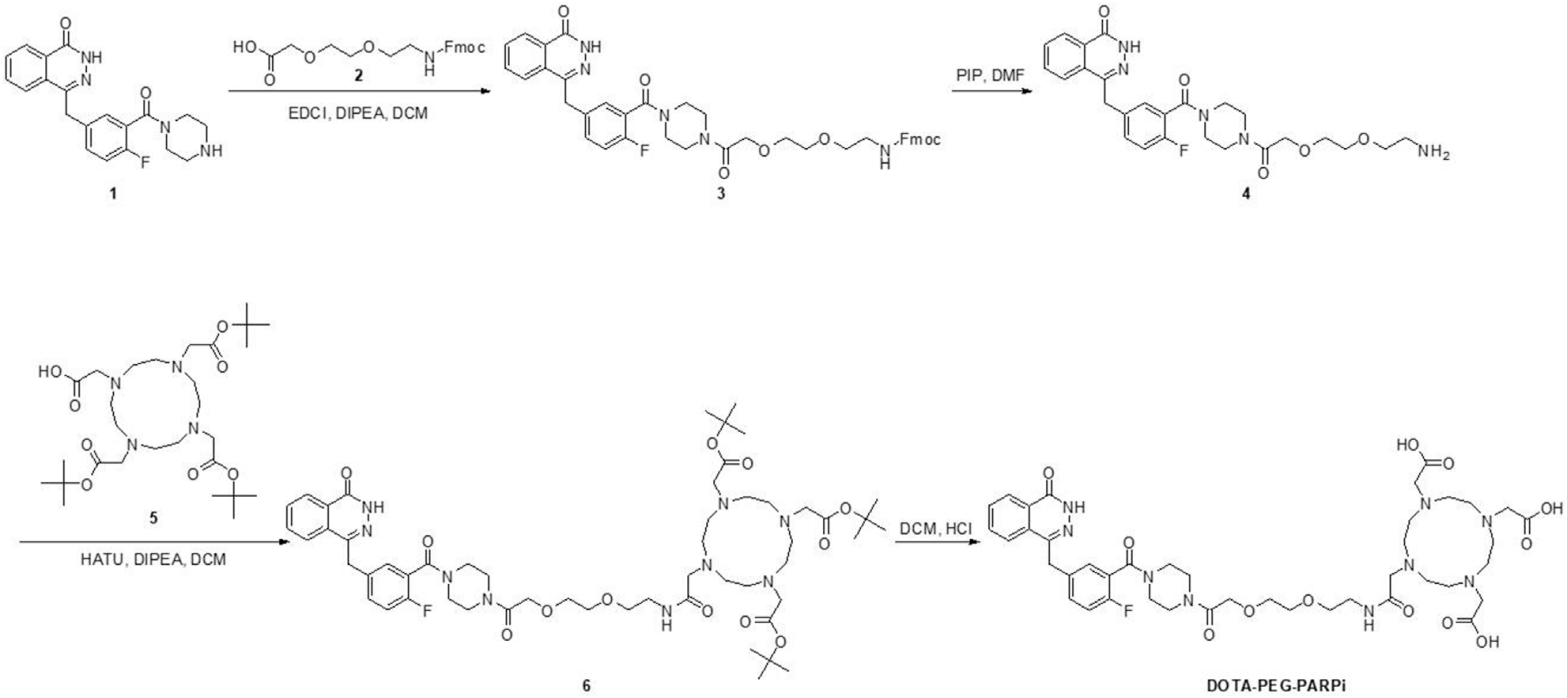
Synthesis of the designed DOTA-PEG-PARPi.

^68^Ga/^177^Lu-DOTA-PEG-PARPi was synthesized, as described in Figure 1. Compound 1 (209.14 mg, 570.82 umol, 1.1 eq), EDCI (129.32 mg, 674.61 umol, 1.3 eq), and DIPEA (257.29 uL, 1.56 mmol, 3 eq) were added to a solution of compound 2 (200 mg, 518.93 umol, 1.0 eq) in DCM (20 mL). The reaction mixture was diluted with DCM and washed with 0.5 M HCl aqueous solution. The aqueous layer was further extracted once with DCM and stirred overnight at room temperature. The combined organic layers were washed with saturated sodium bicarbonate and brine, dried over Na_2_SO_4_, and filtered. The filtrate was concentrated to produce crude compound 3 (568 mg, 99.44%) as a white-foamed solid, which can be used without further purification.

Piperidine (PIP, 2 mL) was added to a solution of compound 3 (568 mg, 774.07 µmol, 1.0 eq) in DMF (8 mL). The reaction mixture was stirred at room temperature for 1.5 h. TLC showed that the reaction was completed. The reaction mixture was washed several times with Et_2_O and PE to yield crude compound 4 (374 mg, 94.45%) as a light yellow oil.

Compound 4 (96.46 mg, 188.57 µmol, 1.2 eq), HATU (89.62 mg, 235.71 µmol, 1.5 eq), and DIPEA (77.91 µL, 471.42 µmol, 3 eq) were added to a solution of compound 5 (90 mg, 157.14 µmol, 1.0 eq) in DCM (4 mL). The reaction mixture was stirred overnight at room temperature. The reaction mixture was diluted with DCM and washed with H_2_O, and the aqueous layer was further extracted once with DCM. The combined organic layers were washed with saturated sodium bicarbonate and brine, dried over Na_2_SO_4_, and filtered. The crude product obtained after vacuum evaporation was purified by column chromatography (DCM: MeOH = 100:1 to 10:1) and Prep-TLC (twice) to yield compound 6 (115 mg, 68.63%) as a white solid.

A measure of 5 M HCl (in 1,4-dioxane) (2 mL) was added to a solution of compound 6 (110 mg, 103.16 umol, 1.0 eq) in DCM (2 mL). The reaction mixture was stirred overnight at room temperature. TLC and HPLC showed that the reaction was completed. The reaction mixture was precipitated with Et_2_O_2_, and the solid was dried to yield **DOTA-PEG-PARPi** (82.3 mg, 88.84%) as a white solid.

^68^GaCl_3_ dissolved in an acetate buffer system (0.5 M, pH = 4.7) was mixed with DOTA-PEG-PARPi at 95°C for 15 min and then passed through an activated C18 column to purify the ^68^Ga-DOTA-PEG-PARPi solution. The solution was diluted with physiological saline, and its radiochemical purity was determined using radio-TLC [NH_4_Ac/MeOH = 1:1 DTPA solution or 0.5 M citric acid buffer (pH5.0), where chromatographic paper is iTLC-SG] and radio-HPLC. The process of ^177^LuCl_3_ radiolabeling is similar to those described above.

### Cell culture and tumor models

A549, AsPC-1, PSN-1, ISN-1, and U87MG cells were purchased from ACTT and cultured as a monolayer using DMEM (Gibco, Carlsbad, United States) and RPMI 1640 medium (Gibco, Carlsbad, United States) containing 10% FBS (Gibco, Carlsbad, United States) and 1% penicillin–streptomycin (Invitrogen, Carlsbad, United States) at 37°C in a humidified atmosphere with 5% CO_2_ and 95% air.

Balb/c nude mice aged 6–8 weeks were purchased from Charles River (Beijing, China) and kept in specific pathogen-free (SPF) conditions during the experimental period. All mice had free access to food and water. All animal experimental procedures were performed in accordance with the guidelines and approved by the Animal Care and Use Committee of the China Institute for Radiation Protection. Following a one-week acclimation period, PSN-1 cells were inoculated subcutaneously into the right flank of the mice (5 × 10^6^ cells/mice) to establish the model. The mice with tumor diameters >1 cm could be used in this experiment.

### ^68^Ga-DOTA-PEG-PARPi *in vitro* binding assays

A549, AsPC-1, PSN-1, ISN-1, and U87MG cells were cultured in 96-well plates for approximately 24 h. To evaluate the binding selectivity of ^68^Ga-DOTA-olaparib and ^68^Ga-DOTA-PEG-PARPi, an unlabeled PARP inhibitor was added at 37°C for 30 min prior to the addition of a ^68^GA-labeled radiotracer. After the removal of the cell culture medium, cells were washed with PBS, and the ^68^Ga content in the cell lysate was determined using a γ-counter.

### ^177^Lu-DOTA-PEG-PARPi *in vitro* cell uptake

A549, AsPC-1, PSN-1, ISN-1, and U87MG cells were seeded in 6-well plates (400 × 10^3^/well) for 24 h. Then, the cells were incubated with ^177^Lu-DOTA-olaparib and ^177^Lu-DOTA-PEG-PARPi (740 kBq/mL in each well), respectively, at 37°C for 2 h, followed by rinsing three times with PBS to eliminate unbound ^177^Lu-labeled radiotracers. Cell suspensions were produced after adding pancreatin (0.5 mL). Finally, the γ-counter was used to measure radioactivity.

### Radiochemical stability

The *in vitro* stability of ^177^Lu-DOTA-PEG-PARPi was evaluated in saline, mouse plasma, and human plasma. In brief, 0.2 mL of ^177^Lu-DOTA-PEG-PARPi (0.74–2.96 MBq) was incubated with 0.8 mL of saline at room temperature for 1 and 24 h and then evaluated by radio-HPLC to assess the stability of ^177^Lu-DOTA-PEG-PARPi. Additionally, ^177^Lu-DOTA-PEG-PARPi (37–74 MBq, 0.2 mL) was added to 0.8 mL of mouse plasma or human plasma, and the solution was incubated at 37°C for 1 and 24 h, respectively. After incubation, 0.2 mL of the above solution was added to 0.4 mL of acetonitrile to precipitate plasma proteins and then swirled. The plasma proteins were subsequently separated by centrifugation at 14,800 rpm for 10 min at 4°C. The supernatant was then analyzed by HPLC. The above experiments were repeated three times.

### Micro PET/SPECT/CT imaging

^68^Ga-DOTA-PEG-PARPi and ^177^Lu-DOTA-PEG-PARPi imaging were obtained using a microPET/SPECT/CT all-in-one machine (Novel Medical, China).

The tumor-bearing mice (n = 1) were intravenously injected with ^68^Ga-DOTA-PEG-PARPi via the lateral tail vein at a dose of 3.7 MBq under isoflurane maintenance anesthesia. A 15-min acquisition was performed at 0.5 and 2 h post-injection. Image reconstruction was performed using the 3D ordered subset expectation maximization (OSEM) algorithm based on the Monte Carlo system model. A CT scan with a tube voltage of 80 kV and a tube current of 0.5 mA was performed on mice after PET acquisition. The image reconstruction was performed based on the filtered back projection (FBP) algorithm. Images were analyzed using NMSoft-AIWS software.

The tumor-bearing mice (n = 3) were intravenously injected with ^177^Lu-DOTA-PEG-PARPi via the lateral tail vein at a dose of 30 MBq under isoflurane maintenance anesthesia. A 30-min acquisition was performed at 1, 3, 6, 24, and 48 h post-injection. Image reconstruction was performed using the 3D OSEM algorithm based on the Monte Carlo system model. A CT scan with a tube voltage of 80 kV and a tube current of 0.5 mA was performed on mice after SPECT acquisition. Image reconstruction was performed based on the FBP algorithm. Images were analyzed using NMSoft-AIWS software. ID%/cc values were obtained by drawing the ROIs of the lesions in the SPECT image and using the automatic thresholding tool.

### *Ex vivo* biodistribution and radiation dosimetry

#### Biodistribution in the model

^177^Lu-DOTA-PEG-PARPi was administered via the tail vein. All mice were euthanized via isoflurane inhalation at 3, 24, 48, 96, and 168 h post-injection, respectively. Blood and organs were collected, dissected, and measured, and the radioactivity was quantified using a γ-counter (2480 WIZARD^2^, PerkinElmer, United States). Organ samples included the pancreas, kidney, heart, liver, spleen, lung, stomach, intestine, bone (femur), muscle, and tumor. Data are expressed as the percentage of the injected dose per gram of tissue (%ID/g).

#### Radiation dosimetry

The mean absorbed radiation doses were evaluated using the mathematic formalism established by MIRD. The estimated dose for an adult human was computed using OLINDA/EXM 2.2.3 (Hermes Medical Solutions, Sweden) with the mice ^177^Lu-DOTA-PEG-PARPi pharmacokinetic data as input. It was assumed that the biodistribution of the tracer in mice was the same as in adult humans. The mice biodistribution data (%ID/g) were converted to human whole-organ biodistribution data (%ID/organ) using formula (a):

$$\left(a\right){\left(\frac{\%\text{ID}}{organ}\right)}_{human}=\left[{\left(\frac{\%\text{ID}}{g_{\text{organ}}}\right)}_{\text{animal}}\times {\left(\text{kgTBweight}\right)}_{\text{animal}}\right]\times {\left(\frac{g_{organ}}{kgTBweight}\right)}_{human}.$$

The calculated total number of decays per gram of tissue was converted to the residence time per gram (MBq*h/MBq/g). Values were then multiplied with the organ mass in the respective male or female MIRD89 phantom to derive the human residence times. The values for human residence times were used as input in OLINDA/EXM.

### Treatment of the pancreatic cancer model

A total of 20 tumor-bearing mice were included and randomly assigned to four groups. The tumor-bearing mice (n = 5) that did not receive treatment were set as the vehicle group (control group). The other three groups of tumor-bearing mice were treated with different doses of ^177^Lu-DOTA-PEG-PARPi (L, low dose; M, medium dose; and H, high dose), that is, a 10 MBq group (n = 5, 10 ± 1.28 MBq), a 20 MBq group (n = 5, 21 ± 1.64 MBq), and a 30 MBq group (n = 5, 29 ± 1.06 MBq). The body weight of these mice was measured twice every week in grams (g) after treatment. The relative body weight (RBW) at day n was calculated according to the formula: RBW = body weight on day n/body weight on day 1. The length (L) and width (W) of the tumor using the same caliper were also recorded in millimeters (mm), and the volume of the tumor was then calculated (V = 0.5 × L × W^2^; mm^3^). The relative TV (RTV) was calculated according to the formula: RTV = TV on day n/TV on day 1. At the end of the study, all mice were euthanized, and the organs and tumors were preserved and tested.

### Statistical analysis

The radioactivity data, as measured using the γ-counter, are presented as CPM with time–decay correction. All experimental results are presented as the mean ± SEM (standard error of the mean). All statistical analyses were performed with GraphPad 9.0 (Prism, United States). ANOVA was employed to examine statistical differences among groups. Survival analysis was conducted using the Kaplan–Meier method to compare the free-of-event rate among the groups. *P*-value <0.05 was considered statistically significant.

## Results

### Radiochemistry

**FIGURE 2:**
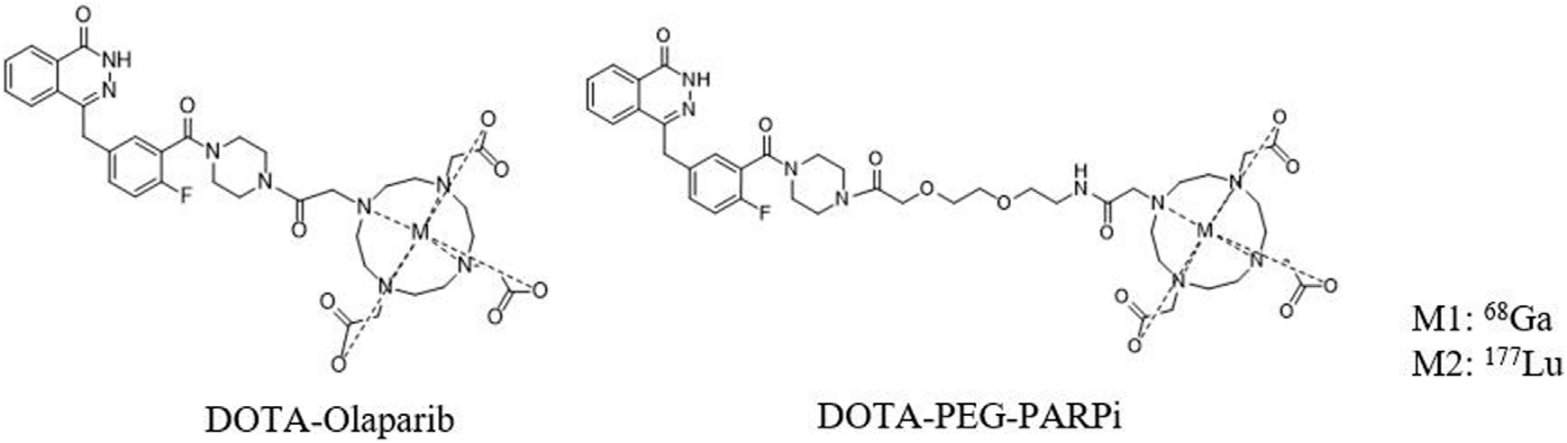
Structure of ^68^Ga/^177^Lu- DOTA-olaparib and DOTA-PEG-PARPi.

The chemical structures of ^68^Ga-DOTA-PEG-PARPi and ^177^Lu-DOTA-PEG-PARPi are shown in Figure 2. Based on radio-HPLC analysis, the radiochemical purity is determined to be more than 95%.

### Binding affinity of ^68^Ga-DOTA-PEG-PARPi

**FIGURE 3:**
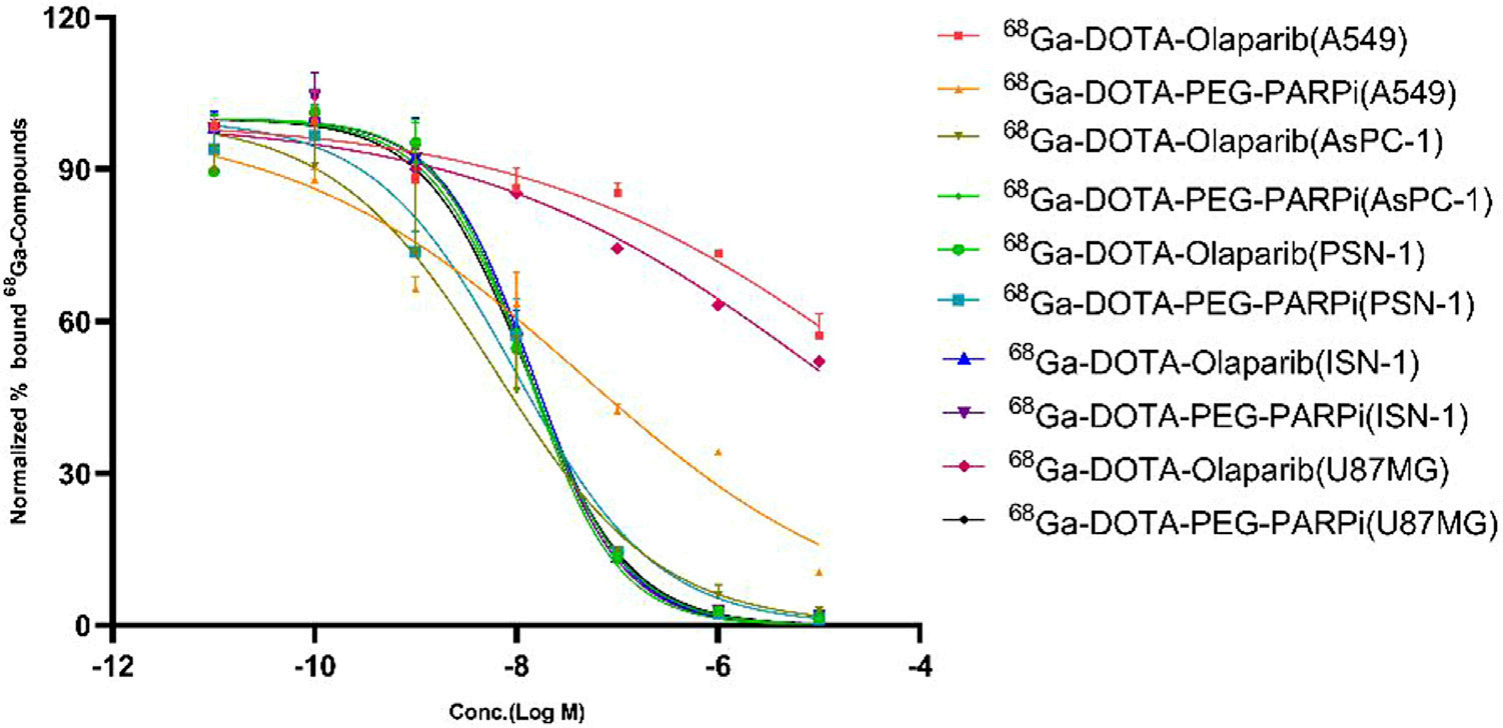
Results of binding affinity and uptake *in vitro* on DOTA-PEG-PARPi.

The binding affinity of ^*68*^ *Ga-*DOTA-olaparib and ^*68*^ *Ga-DOTA-PEG-PARPi* for PARP-1 was determined in A549, AsPC-1, PSN-1, ISN-1, and U87MG cells, the results of which are shown in Figure 3. The IC_50_ values for compounds ^68^Ga-DOTA-olaparib and ^68^Ga-DOTA-PEG-PARPi in PSN-1 were 1.289E-08 and 9.810E-09, respectively. These results indicate that radiotracers have a high affinity in pancreatic cancer toward PARP-1.

### Cell uptake of ^177^Lu-DOTA-PEG-PARPi

**FIGURE 4:**
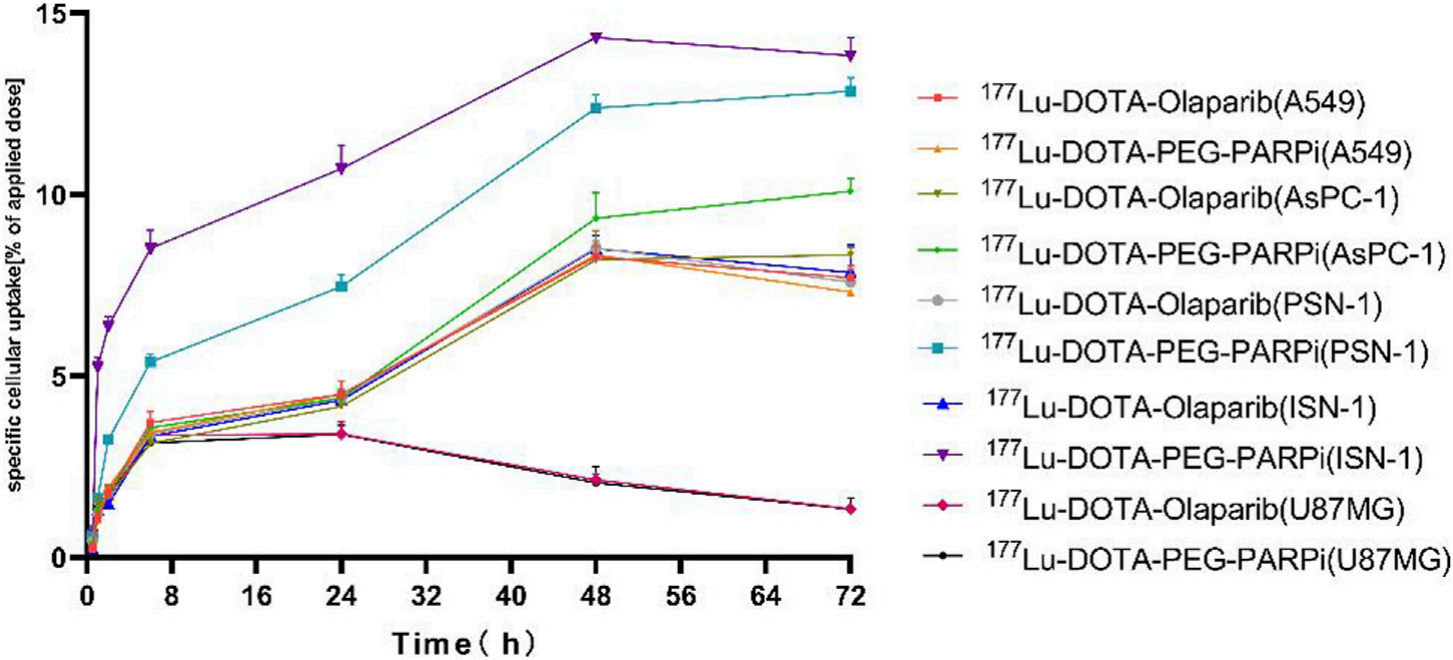
Results of cell uptake *in vitro* on ^177^Lu-DOTA-olaparib and ^177^Lu-DOTA-PEG-PARPi.

As shown in Figure 4, cellular uptake also showed an increasing trend with the increase in time, reaching the maximum at 48 h. The uptake of ^177^Lu-DOTA-PEG-PARPi by A549, AsPC-1, PSN-1, ISN-1, and U87MG cells at 48 h was 9.45%, 13.42%, and 14.34%, and the uptake of ^177^Lu-DOTA-olaparib was 8.34%, 9.36%, 8.21%, 8.54%, and 8.46%, respectively. These results indicate that the radiotracer has high clinical application potential for PARP-1.

### Stability of ^177^Lu-DOTA-PEG-PARPi

**FIGURE 5:**
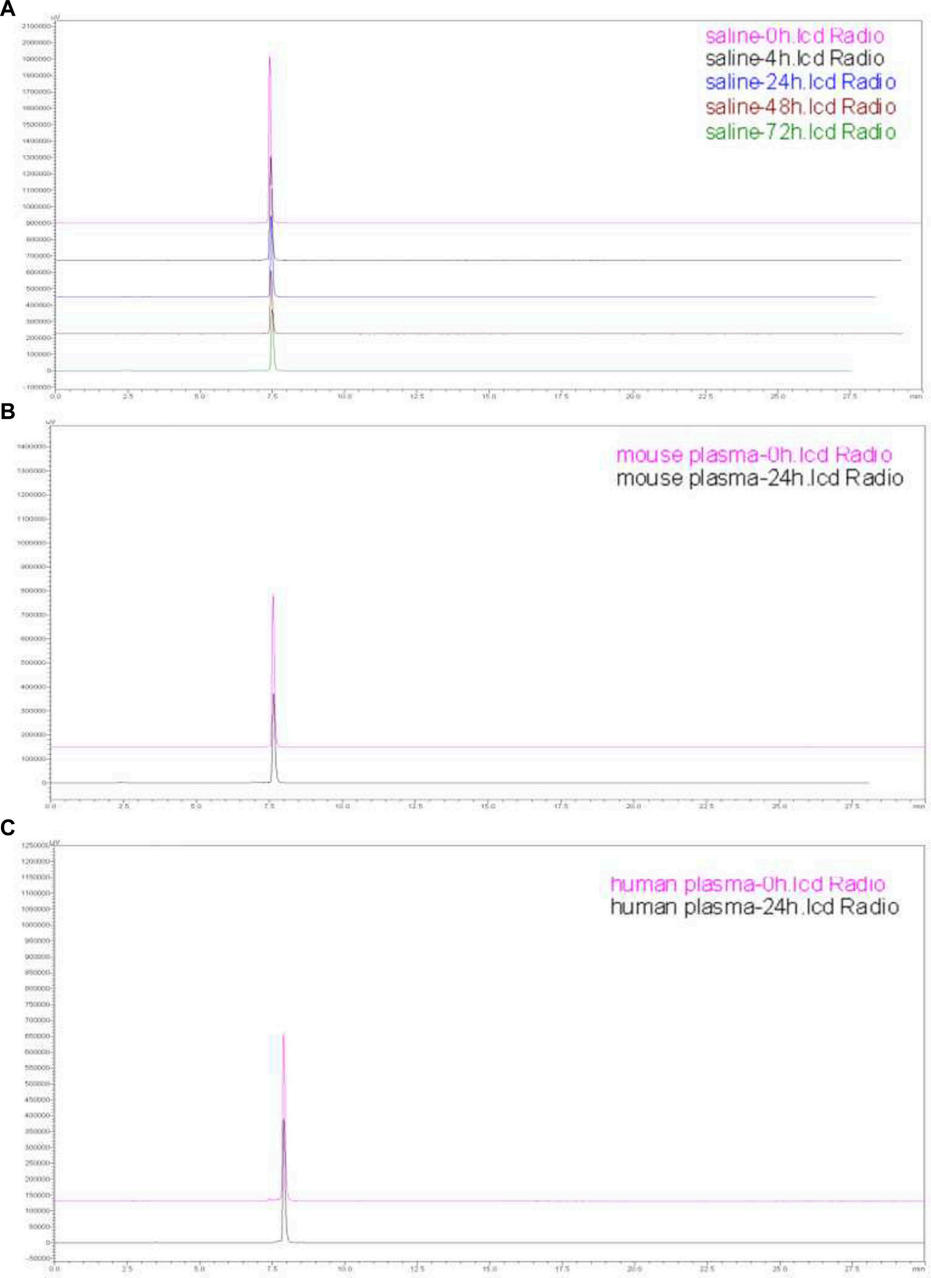
Stability of ^177^Lu-DOTA-PEG-PARPi. Stability of ^177^Lu-DOTA-PEG-PARPi in saline (0, 4, 24, 48, and 72 h) **(A)**, mouse plasma (0 and 24 h) **(B)**, and human plasma (0 and 24 h) **(C)**.

The results exhibited satisfactory *in vitro* stability for ^177^Lu-DOTA-PEG-PARPi at 1 and 24 h, as evaluated by HPLC analysis. As shown in Figure 5, greater than 95% of the prototype radiopharmaceutical had no degradation in saline (after 0, 4, 24, 48, and 72 h) and mouse plasma and human plasma (after 0 and 24 h) incubation. From the *in vitro* stability results, it can be concluded that ^177^Lu-DOTA-PEG-PARPi is stable enough to be used.

### Micro SPECT/CT imaging

**FIGURE 6:**
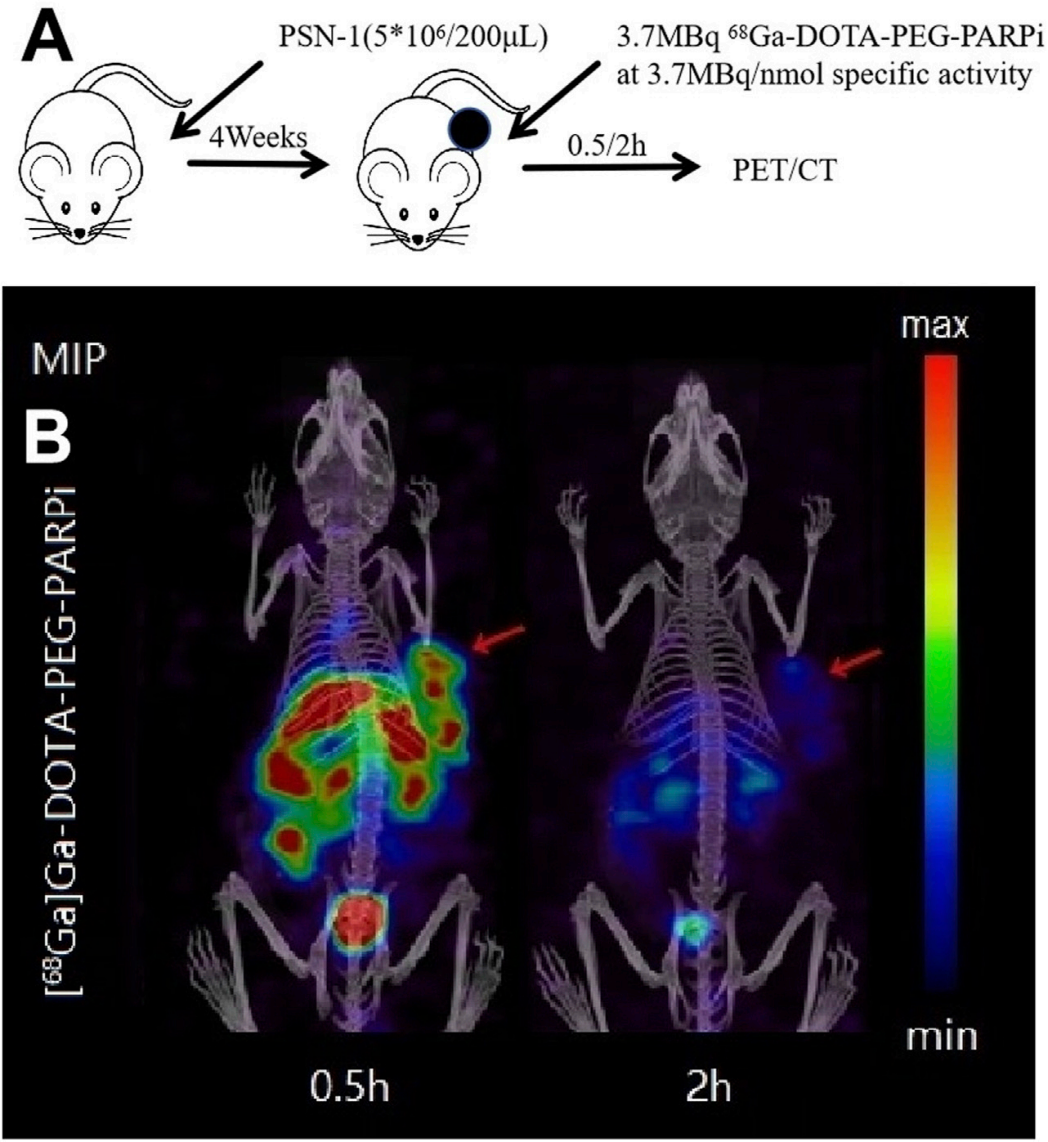
PET/CT result of ^68^Ga-DOTA-PEG-PARPi in PSN-1 models. Molecule imaging of ^68^Ga-DOTA-PEG-PARPi **(A)**. Micro PET/CT images of ^68^Ga-DOTA-PEG-PARPi in PSN-1 models at 0.5, 1, and 2 h after administration **(B)**.

**FIGURE 7:**
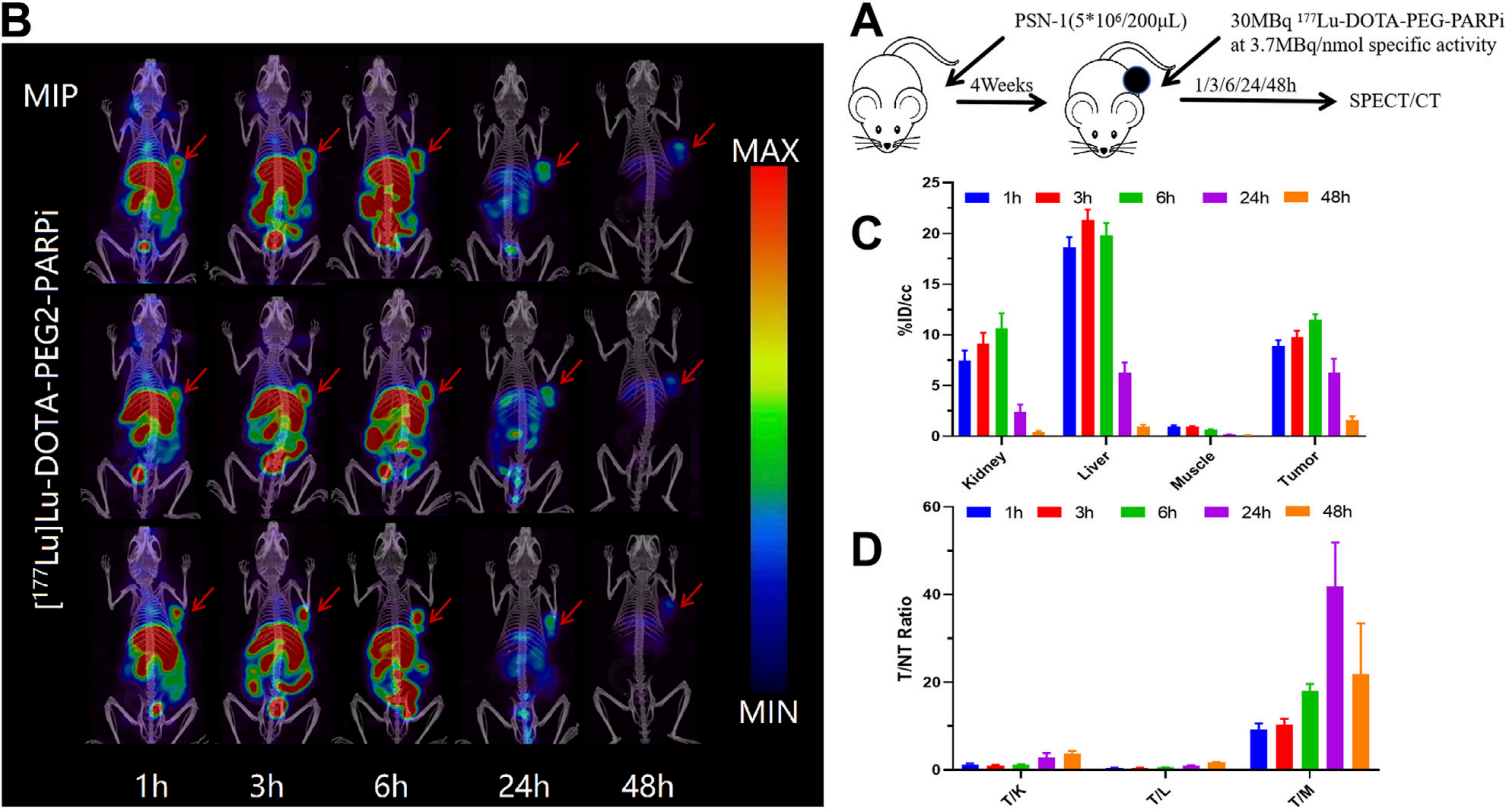
SPECT/CT result of ^177^Lu-DOTA-PEG-PARPi in PSN-1 models. Molecule imaging of ^177^Lu-DOTA-PEG-PARPi **(A)**. Micro SPECT/CT images of ^177^Lu-DOTA-PEG-PARPi in PSN-1 models at 1, 3, 6, 24, and 48 h after administration **(B)**. Ratios of tumor-to-non-target tissue (n = 3). Quantification of the regions of interest (ROIs) in PSN-1 models expressed as %ID/cc **(C)**. T/M (tumor-to-muscle) ratios **(D)**.

^68^Ga-DOTA-PEG-PARPi PET/CT imaging demonstrated significant accumulation in the PSN-1 tumors (Figure 6). SPECT/CT imaging was performed at 48 h after injection of ^177^Lu-DOTA-PEG-PARPi (Figure 7A). ^177^Lu-DOTA-PEG-PARPi SPECT/CT imaging showed a significant concentration of radioactivity in the PSN-1 tumors (Figure 7B), suggesting high uptake and prolonged retention time in the tumor. The uptake of radioactive ^177^Lu-DOTA-PEG-PARPi in the PSN-1 models was approximately 8.88 ± 0.61%ID/cc (1 h), 9.73 ± 0.68%ID/cc (3 h), 11.48 ± 0.56%ID/cc (6 h), 6.29 ± 1.35%ID/cc (24 h), and 1.63 ± 0.38%ID/cc (48 h), as determined by quantitative calculations from the SPECT images. The T/M ratios of ^177^Lu-DOTA-PEG-PARPi were approximately 9.26 ± 1.37 (1 h), 10.37 ± 1.30 (3 h), 17.97 ± 1.67 (6 h), 41.80 ± 10.07 (24 h), and 21.88 ± 11.58 (48 h).

### *Ex vivo* biodistribution of PSN-1 models and dosimetry estimates on adult humans

Female tumor xenograft Balb/c nude mice were randomized into BioD timepoint groups at study day 0 based on body weight. On study day 0, mice were intravenously administered ^177^Lu-DOTA-PEG-PARPi (30 μCi/animal) and euthanized at 3, 24, 48, 96, and 168 h after administration. *Ex vivo* distribution was evaluated by counting 1 min on a γ-counter, and dosimetry was assessed using OLINDA/EXM software.

**FIGURE 8:**
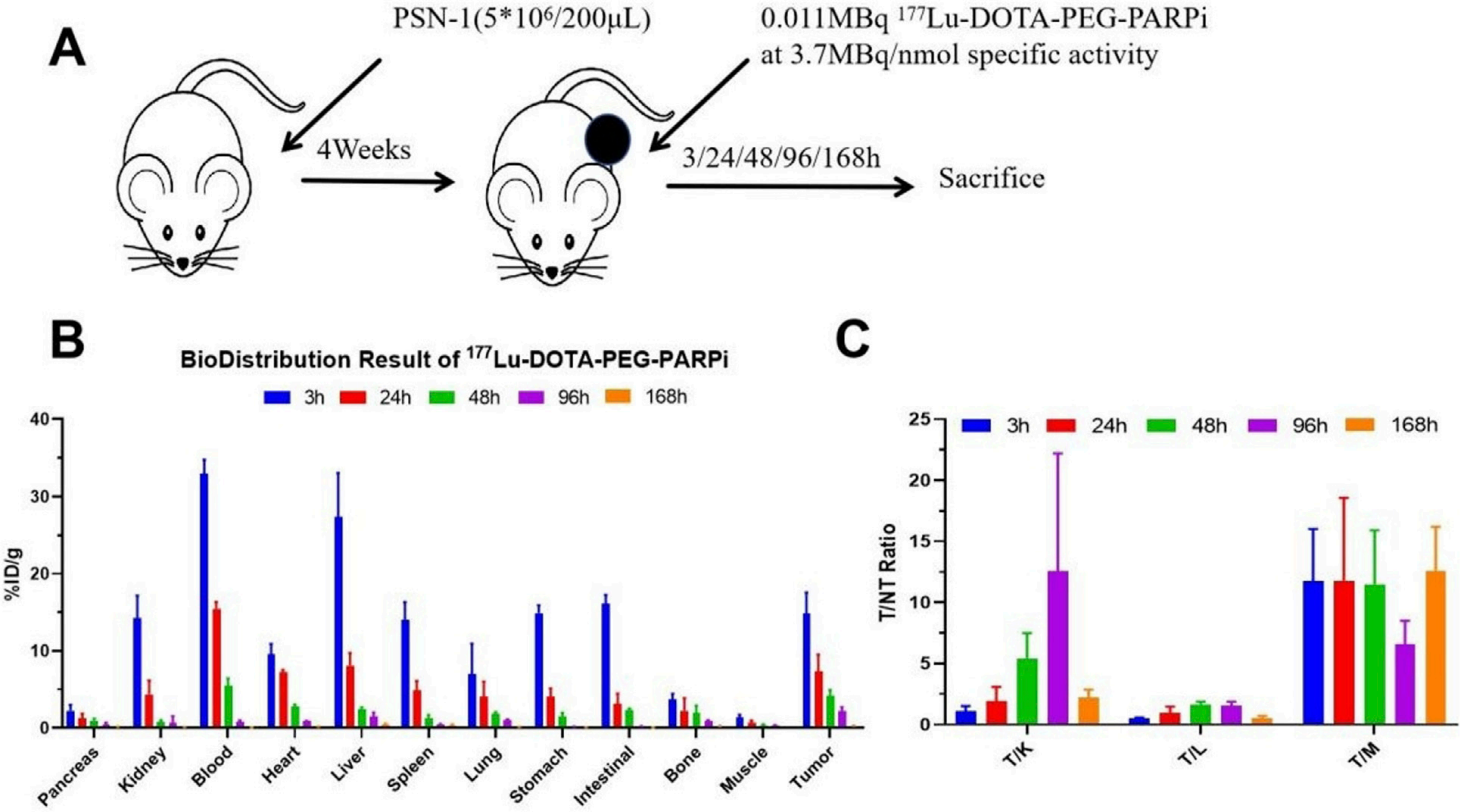
Biodistribution result of 177Lu-DOTA-PEG-PARPi in PSN-1 models. . Biodistribution of 177Lu-DOTAPEG-PARPi in PSN-1 models (*p = 0.046 < 0.05, n = 3) **(A)**. Ratios of tumor-to-non-target tissue (n = 3). Quantification of the regions of interest (ROIs) in PSN-1 models expressed as %ID/g **(B)**. T/M (tumor-to-muscle) ratios **(C)**.

The biodistribution of ^177^Lu-DOTA-PEG-PARPi in PSN-1 tumors and the major organs is shown in Figure 8A. Tumor uptake peaked at 3 h, with an absorption of 102.65% ID/g, and decreased to 40.25% ID/g at 168 h. The liver was the normal organ with the highest uptake, as shown in Figure 8B. Tumor-to-organ uptake ratios of ^177^Lu-DOTA-PEG-PARPi in PSN-1 models are shown in Figure 8C.

As a result, for an adult female (ICRP 103 adult female), the Spleen exhibited the highest absorbed dose (5.52E-03mGy/MBq), followed by the Red Marrow, lung, and Kidneys (4.36E-03, 2.33E-03, and 2.00E-03 mGy/MBq, respectively). The effective dose was 2.00E-02 mSv/MBq (Table 1).

**TABLE 1:** The extrapolated dose estimation data and tumor dose estimation data can be included in the supplementary materials.

Target organ	^177^Lu-DOTA-PEG-PARPi	
	Total (mGy/MBq)	ICRP-103 ED (mSv/MBq)
Adrenals	1.08E-02	9.96E-05
Brain	2.33E-04	2.33E-06
Breasts	4.77E-04	5.72E-05
Esophagus	2.04E-03	8.15E-05
Eyes	2.36E-04	0.00E+00
Gallbladder Wall	1.83E-03	1.69E-05
Left colon	1.33E-02	6.46E-04
Small Intestine	1.23E-02	1.14E-04
Stomach Wall	2.70E-03	3.23E-04
Right colon	1.23E-02	5.97E-04
Rectum	1.17E-02	2.69E-04
Heart Wall	1.00E-01	9.26E-04
Kidneys	2.16E-01	2.00E-03
Liver	4.35E-02	1.74E-03
Lungs	1.94E-02	2.33E-03
Ovaries	4.41E-04	1.76E-05
Pancreas	1.01E-02	9.32E-05
Salivary Glands	1.90E-04	1.90E-06
Red Marrow	3.63E-02	4.36E-03
Osteogenic Cells	7.21E-02	7.21E-04
Spleen	5.98E-01	5.52E-03
Thymus	1.64E-03	1.51E-05
Thyroid	5.17E-04	2.07E-05
Urinary Bladder Wall	2.29E-04	9.17E-06
Uterus	4.16E-04	1.92E-06
Total Body	7.89E-03	0.00E+00
Effective Dose (mSv/MBq)	2.00E-02	

### Effects of ^177^Lu-DOTA-PEG-PARPi treatment on PSN-1 tumor-bearing mice

**FIGURE 9:**
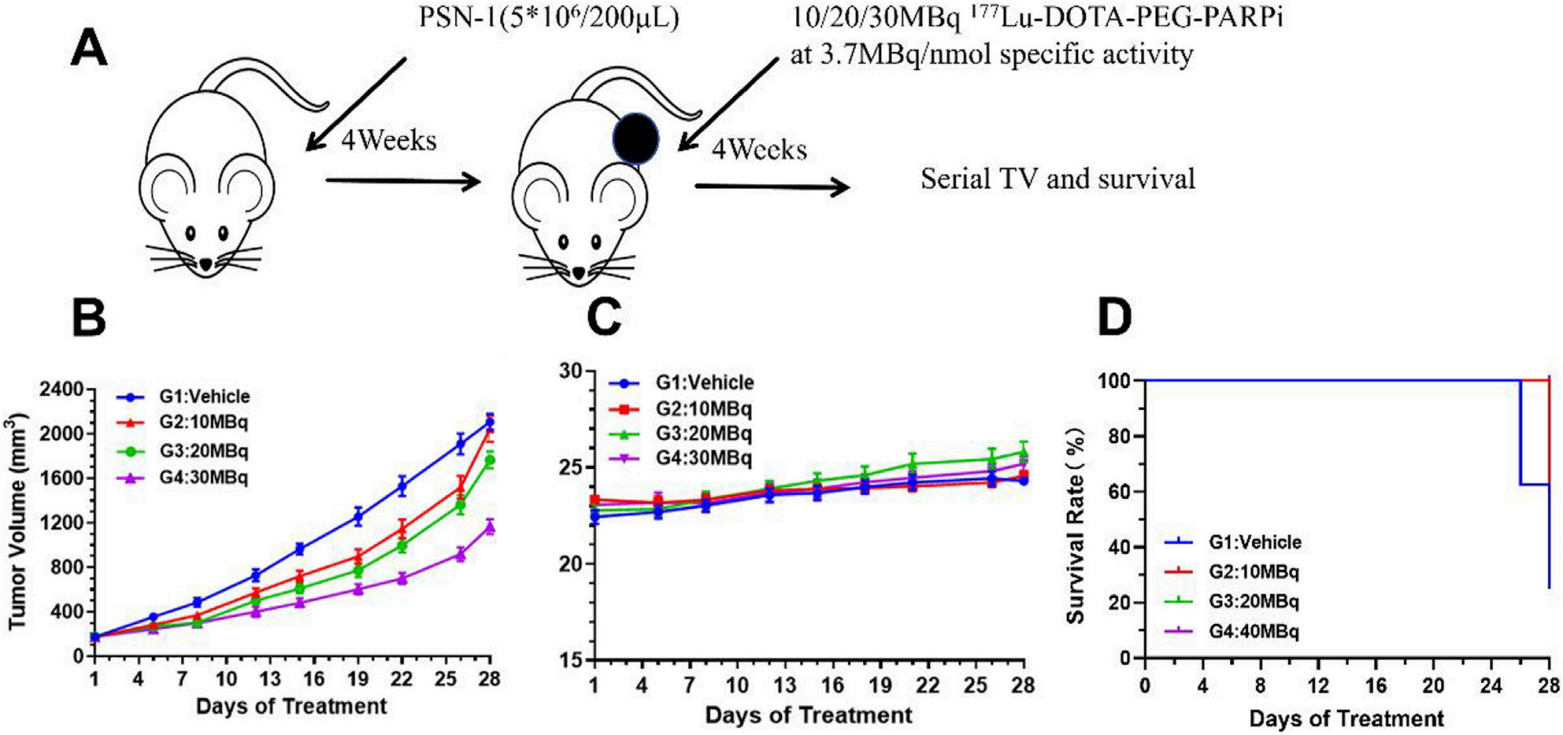
Efficacy result of ^177^Lu-DOTA-PEG-PARPi in PSN-1 models. The effects of ^177^Lu-DOTA-PEG-PARPi treatment on PSN-1 tumor-bearing mice. **(A)** Biodistribution of ^177^Lu-DOTA-PEG-PARPi in PSN-1 tumor-bearing mice (*p < 0.05, n = 3). **(B)** Changes in the relative body weight. **(C)** Changes in the relative lesion size (*p < 0.05, n = 3). **(D)** Kaplan–Meier curves for four groups (*p < 0.05, n = 3).

The *in vivo* activity of ^177^Lu-DOTA-PEG-PARPi was evaluated in a PSN-1 pancreatic cancer xenograft model. Mice were administered ^177^Lu-DOTA-PEG-PARPi via tail vein injection of either vehicle or 10 MBq, 20 MBq, or 30 MBq total activity with a specific activity of 3.7 mBq/nmol (Figure 9A). The mean tumor volume of the vehicle control reached 2,041 mm^3^ after 26 days of treatment (day 28 after tumor inoculation). ^177^Lu-DOTA-PEG-PARPi at 30 mBq/mice as a single dose exhibited moderate antitumor activity, with a final TV of 919 mm^3^ and a TGI value of 51.56%, which were significantly different from those of the vehicle control group (*p* < 0.05). ^177^Lu-DOTA-PEG-PARPi at 20 mBq/mice and 10 mBq/mice as single doses demonstrated lower antitumor activity, with final TVs of 1,365 mm^3^ and 1,521 mm^3^ and TGI values of 28.07% and 19.76%, respectively, which were significantly different from those of the vehicle control group (*p* < 0.05) (Figure 9B). No weight loss was observed in the surviving animals (Figure 9C).

## Discussion

As a key tool for real-time monitoring of the status of tumor DNA damage repair, PARP-1 PET imaging agents have made remarkable progress in recent years in aspects such as the development of novel imaging agents (e.g., [18F]-PARPZ), personalized treatment strategies (screening patients sensitive to PARP inhibitors), and the combination of multimodal imaging techniques. However, their clinical application still faces challenges such as insufficient specificity (non-specific uptake in normal tissues), complexity of clinical translation (interference from tumor heterogeneity), the absence of standardized quantitative approaches, high costs, and limitations in accessibility. In the future, it is essential to optimize the structure of the imaging agents, combine dynamic imaging with AI analysis, and promote multicenter clinical trials to achieve their widespread application in precision cancer treatment (Boinapally et al., 2023; Cao et al., 2023; Kong et al., 2022; Kostos et al., 2022; Zhang et al., 2024; Zheng et al., 2023).

In the study, we used the PARP-1 inhibitor olaparib as the parent structure to successfully synthesize a novel probe capable of chelating medical radiometals (^68^Ga and ^177^Lu). Its potential as a targeted PARP-1 probe in diagnosis and treatment was evaluated through *in vitro* and *in vivo* druggability studies. The objective of our study is to develop a PARP-1 theranostic probe with clinical application potential, providing tools for patient screening, therapeutic response prediction, quantitative evaluation of therapeutic effect, and new therapeutic approaches directly targeting PARP-1. To address the instability issues associated with ^18^F-labeled PARPi probes *in vivo*, this study utilized the radiometals ^68^Ga and ^177^Lu to label PARPi. These two radionuclides exhibit excellent *in vivo* stability and suitable physical properties, with ^68^Ga emitting positrons suitable for PET imaging and ^177^Lu-emitting β-rays suitable for radiotherapy. This labeling strategy aims to develop a PARPi probe with high stability and strong affinity, exploring its clinical application potential.

Additionally, polyethylene glycol (PEG) modification was employed during the labeling process to optimize the physicochemical properties of the probe. PEG modification enhances the water solubility of the probe and reduces immunogenicity and non-specific protein binding, thereby decreasing the liver and spleen uptake and improving the probe biodistribution and tumor-targeting specificity. The experimental results demonstrated that the PEG-modified PARPi probe not only retained very high affinity (IC_50_ = 6.491E-09) but also significantly reduced liver accumulation *in vivo*, which is critical for minimizing potential side effects and improving therapeutic safety. To evaluate the efficacy and safety of the novel PARPi probes *in vivo*, tumor models were established by transplanting these cell lines into immunodeficient mice. These mice were then administered ^177^Lu- and ^68^Ga-labeled PARPi probes, and changes in tumor size and body weight were monitored to assess the probes’ tumor-inhibitory effects *in vivo*. The results showed that both labeled PARPi probes exhibited significant tumor growth inhibition, with the ^177^Lu-labeled probe demonstrating greater therapeutic potential. In summary, this study developed a novel, stable, and effective PARP-1-targeted probe and validated its potential therapeutic effects in animal models. Future research will focus on further optimizing the probe design to enhance safety and efficacy, ultimately advancing toward clinical application.

Despite the significant progress achieved in this study, several limitations remain. First, the *in vitro* experiments were conducted with a limited sample size, and key characterizations, such as pharmacokinetics, were not thoroughly analyzed in relation to the drug structure and lipid–water partition coefficients. Second, comprehensive toxicity studies were not conducted, and the maximum tolerated dose (MTD) in animals was not determined, leaving the safety assessment incomplete. Finally, since animal models cannot fully replicate the conditions of pancreatic cancer patients, particularly when pancreatic lesions are located near the liver, β-emitting radionuclides may cause crossfire effects on liver tissue. Consequently, the actual liver dose might have been underestimated in animal experiments, and the available dose estimates may not provide comprehensive information for clinical translation. These challenges need to be addressed in subsequent studies to facilitate the clinical application of PARP-1-targeted probes. With continuous optimization and refinement, it is anticipated that PARP-1-targeted probes can be developed into a precise and effective tool to benefit cancer patients.

## Conclusion

In the study, we introduced a chelating group into the lead compound 4-(4-fluoro-3-(piperazine-1-carbonyl)benzyl)phthalazin-1(2H)-one, thus obtaining olaparib derivatives capable of chelating diagnostic and therapeutic radionuclides (gallium-68 and lutetium-177). After radionuclide labeling, a diagnostic and therapeutic probe targeting PARP-1 was obtained. The results showed that lutetium-177-DOTA-polyethylene glycol-PARPi (^177^Lu-DOTA-PEG-PARPi), gallium-68-DOTA-polyethylene glycol-PARPi (^68^Ga-DOTA-PEG-PARPi), and polyethylene glycol-PARPi (PEG-PARPi) had high chemical yields, radiochemical yields, and purities. The imaging results demonstrated that ^68^Ga-DOTA-PEG-PARPi showed significant accumulation in PSN-1 tumors, while ^177^Lu-DOTA-PEG-PARPi had significant uptake in tumors and a prolonged retention time. This study has demonstrated that PEG-PARPi has the potential to be a promising radiotracer for further clinical applications and, ultimately, for the personalized treatment of pancreatic cancer patients.

## Data Availability

The original contributions presented in the study are included in the article/supplementary material; further inquiries can be directed to the corresponding author.

## References

[B1] Bhamidipati D. Haro-Silerio J. I. Yap T. A. Ngoi N. (2023). PARP inhibitors: enhancing efficacy through rational combinations. Br. J. Cancer 129 (6), 904–916. 10.1038/s41416-023-02326-7 37430137 PMC10491787

[B2] Bhin J. Paes Dias M. Gogola E. Rolfs F. Piersma S. R. de Bruijn R. (2023). Multi-omics analysis reveals distinct non-reversion mechanisms of PARPi resistance in BRCA1-versus BRCA2-deficient mammary tumors. Cell Rep. 42 (5), 112538. 10.1016/j.celrep.2023.112538 37209095 PMC10242444

[B3] Boinapally S. Alati S. Jiang Z. Yan Y. Lisok A. Singh R. (2023). Preclinical evaluation of a new series of albumin-binding (177)Lu-labeled PSMA-based low-molecular-weight radiotherapeutics. Molecules 28 (16), 6158. 10.3390/molecules28166158 37630410 PMC10459686

[B4] Cao Q. Li L. Zhao Y. Wang C. Shi Y. Tao X. (2023). PARPi decreased primary ovarian cancer organoid growth through early apoptosis and base excision repair pathway. Cell Transpl. 32, 9636897231187996. 10.1177/09636897231187996 PMC10369085 37488947

[B5] Chen M. Linstra R. Van Vugt M. (2022). Genomic instability, inflammatory signaling and response to cancer immunotherapy. Biochim. Biophys. Acta Rev. Cancer 1877 (1), 188661. 10.1016/j.bbcan.2021.188661 34800547

[B6] Colombo I. Lheureux S. Oza A. M. (2018). Rucaparib: a novel PARP inhibitor for BRCA advanced ovarian cancer. Drug Des. Devel Ther. 12, 605–617. 10.2147/DDDT.S130809 PMC5868608 29606854

[B7] Cortesi L. Rugo H. S. Jackisch C. (2021). An overview of PARP inhibitors for the treatment of breast cancer. Target Oncol. 16 (3), 255–282. 10.1007/s11523-021-00796-4 33710534 PMC8105250

[B8] Dibitetto D. Widmer C. A. Rottenberg S. (2024). PARPi, BRCA, and gaps: controversies and future research. Trends Cancer 10 (9), 857–869. 10.1016/j.trecan.2024.06.008 39004561

[B9] Ke B. Li A. Fu H. Kong C. Liu T. Zhu Q. (2022). PARP-1 inhibitors enhance the chemosensitivity of leukemia cells by attenuating NF-кB pathway activity and DNA damage response induced by Idarubicin. Acta Biochim. Biophys. Sin. (Shanghai) 54 (1), 91–98. 10.3724/abbs.2021011 PMC9909352 35130631

[B10] Kong L. Xu J. Yu L. Liu S. Liu Z. Xiang J. (2022). Construction of PARPi resistance-related competing endogenous RNA network. Curr. Genomics 23 (4), 262–274. 10.2174/1389202923666220527114108 36777878 PMC9875538

[B11] Kostos L. Buteau J. P. Yeung T. Iulio J. D. Xie J. Cardin A. (2022). AlphaBet: combination of Radium-223 and [(17) (7)Lu]Lu-PSMA-I&T in men with metastatic castration-resistant prostate cancer (clinical trial protocol). Front. Med. (Lausanne) 9, 1059122. 10.3389/fmed.2022.1059122 36465905 PMC9716623

[B12] Li W. H. Wang F. Song G. Y. Yu Q. H. Du R. P. Xu P. (2023). PARP-1: a critical regulator in radioprotection and radiotherapy-mechanisms, challenges, and therapeutic opportunities. Front. Pharmacol. 14, 1198948. 10.3389/fphar.2023.1198948 37351512 PMC10283042

[B13] Mccann K. E. Hurvitz S. A. (2018). Advances in the use of PARP inhibitor therapy for breast cancer. Drugs Context 7, 212540. 10.7573/dic.212540 30116283 PMC6089618

[B14] Mcdonald E. S. Pantel A. R. Shah P. D. Farwell M. D. Clark A. S. Doot R. K. (2021). *In vivo* visualization of PARP inhibitor pharmacodynamics. JCI Insight 6 (8), e146592. 10.1172/jci.insight.146592 33884961 PMC8119179

[B15] Reiner T. Keliher E. J. Earley S. Marinelli B. Weissleder R. (2011). Synthesis and *in vivo* imaging of a 18F-labeled PARP1 inhibitor using a chemically orthogonal scavenger-assisted high-performance method. Angew. Chem. Int. Ed. Engl. 50 (8), 1922–1925. 10.1002/anie.201006579 21328671 PMC3471140

[B16] Safdar R. Mishra A. Shah G. M. Ashraf M. Z. (2024). Poly (ADP-ribose) Polymerase-1 modulations in the genesis of thrombosis. J. Thromb. Thrombolysis 57 (5), 743–753. 10.1007/s11239-024-02974-3 38787496

[B17] Slade D. (2020). PARP and PARG inhibitors in cancer treatment. Genes Dev. 34 (5-6), 360–394. 10.1101/gad.334516.119 32029455 PMC7050487

[B18] Soung Y. H. Chung J. (2023). Combination treatment strategies to overcome PARP inhibitor resistance. Biomolecules 13 (10), 1480. 10.3390/biom13101480 37892162 PMC10604269

[B19] Steffen J. D. Tholey R. M. Langelier M. F. Planck J. L. Schiewer M. J. Lal S. (2014). Targeting PARP-1 allosteric regulation offers therapeutic potential against cancer. Cancer Res. 74 (1), 31–37. 10.1158/0008-5472.CAN-13-1701 24189460 PMC3903668

[B20] Wang X. Liu W. Li K. Chen K. He S. Zhang J. (2023). PET imaging of PARP expression using (68)Ga-labelled inhibitors. Eur. J. Nucl. Med. Mol. Imaging 50 (9), 2606–2620. 10.1007/s00259-023-06249-6 37145164 PMC10317875

[B21] Wang Y. Luo W. Wang Y. (2019). PARP-1 and its associated nucleases in DNA damage response. DNA Repair (Amst) 81, 102651. 10.1016/j.dnarep.2019.102651 31302005 PMC6764844

[B22] Young A. J. Pantel A. R. Kiani M. Doot R. K. Bagheri S. Pryma D. A. (2024). Kinetic analysis and metabolism of poly(adenosine diphosphate-ribose) polymerase-1-targeted (18)F-fluorthanatrace PET in breast cancer. J. Nucl. Med. 65, 1862–1868. 10.2967/jnumed.124.268254 39477499 PMC11619586

[B23] Zhang J. Lin Y. Gao J. Pan Y. Hou G. Guo C. (2024). Development and biological evaluation of (68)Ga-labeled peptides for potential application in HER2-positive colorectal cancer. Bioorg Chem. 151, 107645. 10.1016/j.bioorg.2024.107645 39059074

[B24] Zheng Y. Long T. Peng N. Zhen M. Ye Q. Zhang Z. (2023). The value of targeting CXCR4 with 68Ga-pentixafor PET/CT for subtyping primary aldosteronism. J. Clin. Endocrinol. Metab. 109 (1), 171–182. 10.1210/clinem/dgad421 37477496

[B25] Zhou D. Xu J. Mpoy C. Chu W. Kim S. H. Li H. (2018). Preliminary evaluation of a novel (18)F-labeled PARP-1 ligand for PET imaging of PARP-1 expression in prostate cancer. Nucl. Med. Biol. 66, 26–31. 10.1016/j.nucmedbio.2018.08.003 30195072 PMC6252111

